# The dual role of azoles: lifesaving antifungals and drivers of resistance – a One Health perspective

**DOI:** 10.1038/s41467-026-71762-9

**Published:** 2026-04-19

**Authors:** Behrad Roohi, Joel D. A. Tyndall, Thomas Svoboda, Sybren de Hoog, Pedro W. Crous, Brian C. Monk, Birgit Strobl, Annemarie Weissenbacher, Joseph Strauss, Paul E. Verweij, Michaela Lackner

**Affiliations:** 1 Department of Visceral, Transplant and Thoracic Surgery, Medical University of Innsbruck, Innsbruck, Austria; 2 MYCOS – PhD Training Program, Medical University of Innsbruck, Innsbruck, Austria; 3 School of Pharmacy, University of Otago, Dunedin, New Zealand; 4 Institute of Microbial Genetics, Department of Agricultural Sciences, BOKU University Vienna, Campus Tulln, Tulln, Austria; 5 Department of Medical Microbiology and Radboudumc–CWZ Center of Expertise for Mycology, Radboud University Medical Center, Nijmegen, the Netherlands; 6 Westerdijk Fungal Biodiversity Institute, Utrecht, the Netherlands; 7 Sir John Walsh Research Institute, School of Dentistry, University of Otago, Dunedin, New Zealand; 8 Centre of Biological Sciences, University of Veterinary Medicine Vienna, Vienna, Austria; 9 BMOSA – BOKU Corefacility for Bioactive Metabolites Screening and Analysis, BOKU University Vienna, Campus Tulln, Tulln, Austria; 10 Center for Infectious Disease Research, Diagnostics and Laboratory Surveillance, National Institute for Public Health and the Environment (RIVM), Bilthoven, the Netherlands; 11 FOCUS-AµR — Fungal One Health Coalition for Universal Strategies to contain AntiMycotic Resistance, ISHAM/ECCM Working Group, Innsbruck, Austria; 12 Institute of Hygiene and Medical Microbiology, Medical University of Innsbruck, Innsbruck, Austria

**Keywords:** Policy and public health in microbiology, Antimicrobial resistance, Antifungal agents

## Abstract

Azole antifungals are essential for controlling fungal diseases in medicine, veterinary care and agriculture. However, extensive cross-sector use has accelerated the emergence of resistant fungal pathogens, threatening human health, food security and ecosystem stability. This Review examines the dual role of azoles as life-saving therapeutics and drivers of antifungal resistance. We outline their development, mechanisms of action and applications across sectors, and highlight environmental and evolutionary pressures shaping resistance. Integrating perspectives from microbiology, agriculture and public health, we argue that coordinated One Health stewardship and sustainable antifungal strategies are urgently needed to preserve the efficacy of these critical compounds.

## Abstract (web summary)

The same azole antifungals that protect patients and crops are driving resistance across ecosystems. This Review explores how cross-sector use is reshaping fungal evolution and outlines One Health strategies to safeguard antifungal effectiveness.

## Introduction

Azoles are indispensable antifungal agents across human medicine, veterinary care, and crop protection. Their broad-spectrum activity and favourable safety profiles have transformed outcomes for patients at risk of invasive fungal disease and protected yields in high-value and staple crops under intensifying biotic and climatic pressures. This dual reality, where essential utility coexists with selection for antifungal resistance, now spans One Health interfaces. In plants, resistance evolution has reduced azole sensitivity in major pathogens, such as *Pseudocercospora fijiensis* and *Zymoseptoria tritici*. In humans, environmental selection has contributed to azole-resistant *Aspergillus fumigatus* impacting clinical care. Addressing azole stewardship within an integrated One Health framework is therefore critical to sustain efficacy across sectors.

Azoles [demethylation inhibitors (DMIs), FRAC group 3]^1^ uniquely combine broad, systemic, and durable control of fungal pathogens in crops with potent, orally bioavailable, relatively safe first-line therapy in human medicine. Each is anchored in inhibition of sterol 14α-demethylase (SDM; CYP51, also known as Erg11 in *Saccharomyces cerevisiae*) and thus effective across diverse fungi. This efficacy across diverse fungal taxa, favourable pharmacology, scalable manufacturing, and cost-effectiveness has no like-for-like substitutes: agricultural alternatives lack the same spectrum/systemicity and are often more resistance-prone or agronomically constrained, while medical alternatives (e.g., echinocandins, polyenes, newer agents) are limited by route of administration, toxicity, spectrum gaps, or access. However, widespread azole use has imposed significant selection pressure, leading to the emergence and, in some cases, dominance of resistant fungal populations. This phenomenon is observed not only in phytopathogenic fungi but also in medically significant fungi. Azole resistance, increasingly driven by both clinical and environmental selection pressures, poses a growing threat to the long-term efficacy of azoles, raising serious concerns for global health and food security^2,3^.

A recent report from the European Food Safety Authority (EFSA) highlights the problem of azole resistance selection in aspergilli from a One Health perspective. The report notes that azole sales for non-medical purposes across Europe amount to approximately 10,000 tonnes annually. Outside human medicine, azoles are used to treat fungal diseases in animals (veterinary medicinal products, VMPs), protect plants (plant protection products, PPPs), and preserve materials (biocidal products, BPs). Additionally, azoles are sold as industrial chemicals (e.g., intermediates or dyes) and are used in cosmetics, such as anti-dandruff shampoos. Notably, azole PPPs account for over 99% of total azole sales volume (in weight) in the European Union, while human and veterinary medicine together represent only 0.02%^4,5^.

In medicine, the number of individuals who are immunosuppressed or immunocompromised due to conditions, such as HIV infection, cancer, stem cell or organ transplants, and autoimmune disease, is estimated to exceed 100–150 million^6,7^. These patients are at high risk of developing severe, often life-threatening fungal infections. At the same time, PPPs are critical for ensuring the food supply for a global population now exceeding 8 billion, in a context where plant diseases contribute to approximately 30% of global crop losses^8,9^.

Despite decades of use, treatment options for deep-seated fungal infections in human and veterinary medicine remain limited to azoles, echinocandins, and polyenes. Novel antifungals with new mechanisms of action (MoAs) against eukaryotic pathogens, such as fungi are challenging to develop. On the other hand, azoles remain a cornerstone of crop protection because they uniquely combine pan-fungal, systemic, protectant-and-curative activity across a diverse range of plant pathogens, and they stabilize resistance management by partnering in mixtures with other agricultural fungicides, such as quinone outside inhibitors (QoIs) [FRAC 11], quinone inside inhibitors (QiIs) [FRAC 21], β-tubulin inhibitors (MBCs) [FRAC 1] or succinate dehydrogenase inhibitors (SDHIs) [FRAC 7] (Supplementary Table 1)^1,10–13^.

**Fig. 1:**
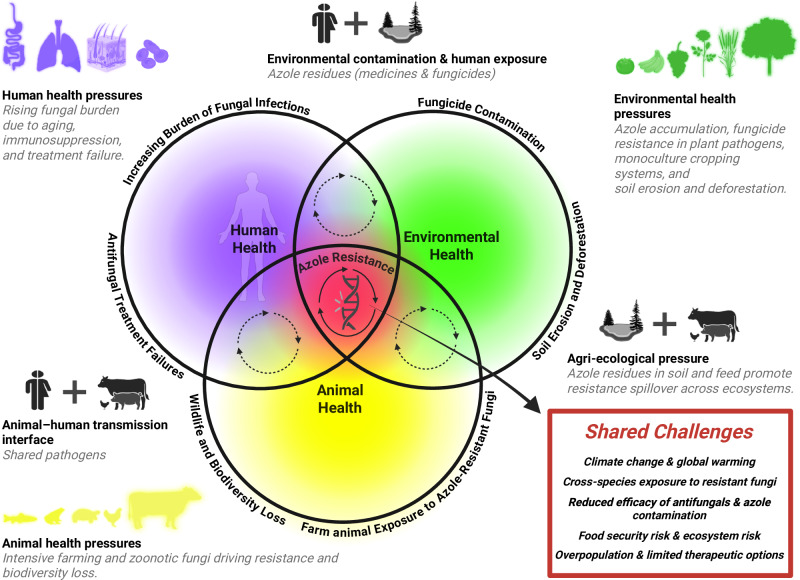
One Health drivers and consequences of azole resistance across human, animal, and environmental sectors. Conceptual framework illustrating how azole use across medicine, veterinary care, and agriculture generates interconnected selection pressures that drive antifungal resistance across One Health domains. Environmental contamination by azole residues from medical and agricultural sources creates shared exposure interfaces linking human, animal, and environmental reservoirs. Resistant fungal populations emerging in environmental or agricultural niches can spread across sectors through ecological interfaces, including wildlife, livestock, plant systems, and human contact with contaminated environments. These interconnected pathways amplify resistance selection and facilitate cross-sector transmission of azole-resistant fungi. The resulting feedback loops contribute to treatment failures, agricultural disease management challenges, and broader ecosystem impacts, highlighting azole resistance as a global One Health problem requiring coordinated stewardship across sectors. Created in BioRender. ROOHI, B. (2026) https://BioRender.com/sx23m3l.

The strong cross-sectoral reliance on azoles comes at a cost. Azole use exerts selection pressure that accelerates the development and spread of resistant fungal populations, which can transfer between environmental, agricultural, veterinary, and clinical settings. These impacts illustrate how resistance has become a global multi-sectoral issue^5^. The fundamental problem of multiple-use is that the applied compounds are structurally similar and have identical MoA (FRAC class), creating selection pressure for azole-resistant phyto-, human- and animal-pathogenic fungi. Molecular resistance patterns in the target gene (SDM) are highly preserved between pathogenic fungi, and can occur as acquired or intrinsic resistance^14^. Most human-pathogenic fungi are opportunistic pathogens, living as saprobes in organic waste piles (e.g., compost, wood waste). These niches serve as selection hotspots for the evolution of resistant *A. fumigatus*. The large-scale use of azoles poses a major risk for the selection of diverse resistant clones, the evolution of new drug-resistant species (intrinsic and acquired), or both, which may subsequently cause fungal diseases across sectors affecting wildlife, pets, livestock, crops, plants, and humans. Therefore, azole contamination and azole resistance presents a One Health challenge, with impacts rippling through interconnected domains of human, animal, and environmental health (Fig. 1)^4^.

Resistant plant pathogenic fungal strains undermine crop protection strategies, while cross-resistance in non-target human fungal pathogens severely limits treatment options in human and veterinary medicine, creating cascading effects that jeopardize ecosystem balance and food security^15^. Acknowledging this, substantial recent global initiatives, such as detailed discussions by G7 Health Ministers, have stressed the important need for collaborative, cross-sectoral strategies to combat antimicrobial resistance (AMR)^16^, including antifungal resistance (AFR).

Although precise global surveillance data on antifungal resistance–attributable deaths are lacking, evidence indicates that resistance is driving a growing proportion of fungal infection–related mortality^17^. Recent estimates suggest that invasive fungal infections cause approximately 2.55 million deaths annually^17^, a burden surpassing AMR bacterial infections, which caused 1.27 million deaths in 2019^18^.

This review synthesizes current evidence on azole resistance, the environmental ramifications of widespread azole use, and the broader health and economic impacts across sectors. Framed within a One Health paradigm, this work aims to distill actionable insights for sustainable azole stewardship that safeguards agricultural productivity while protecting public health.

## Phyto-, animal-, human- and opportunistic fungi

### Phyto-pathogenic fungi - key crops under fungal threat

Cereals (wheat, rice, maize), tubers (potato, cassava, sweet potato, yam), and pulses (soybean, lentil, chickpea, dry beans) underpin the global food supply, together with fruits and vegetables essential for dietary quality. Monoculture-based intensification heightens vulnerability to fungal disease from field to storage. Fungi and storage pests account for up to 30% of annual harvest losses—approximately 10–20% during production and ~10% post-harvest—imposing major economic burdens and worsening food insecurity, particularly across the global South^8,19^. Losses are typically lower in high-income settings but can reach 40% in some low-income contexts, reflecting structural inequities in access to context-appropriate pest management, harvesting and processing technologies, storage infrastructure, and management support, rather than inherent differences in fungal pressure^20,21^. These losses erode yields and the economic and nutritional stability of legume and oilseed systems. Crops, such as soybean, chickpea, and oilseed rape face persistent fungal threats that depress yields and quality. Fruits and vegetables are similarly affected, with humidity and temperature further accelerating fungal growth and shortening post-harvest longevity^22,23^. Major cereal pathogens—including wheat stem rust (*Puccinia graminis*), Fusarium head blight (FHB), rice blast (*Pyricularia oryzae*), powdery mildew (*Blumeria graminis*), and northern corn leaf blight (*Exserohilum turcicum*)—recurrently depress yields and quality. Uncontrolled stem rust can cut wheat yields by 10–90% and has periodically disrupted global supply^19^. Rice blast remains globally destructive, with outbreaks in Kenya in the 2020 s driving regional production shortfalls of up to 48%^24^. In maize, northern corn leaf blight has caused major episodic losses, including an estimated 132 million bushels in the U.S. Corn Belt in 2013. These diseases exploit favourable weather and genetic uniformity, underscoring the need for integrated crop management and durable resistance to fungal pathogens^25^.

Trees underpin terrestrial ecosystems through carbon sequestration, oxygen production, and habitat provision, yet fungal pathogens increasingly threaten forest health and biodiversity, causing widespread tree mortality with significant ecological and economic consequences. These impacts extend beyond forests, affecting timber industries, ecosystem resilience, and climate adaptability^26^. Comparable pressures in horticulture depress yields and quality, influence consumer demand and market access, and trigger trade restrictions. Climate change is poised to amplify disease prevalence and severity. Although fungicides remain essential, disease management still relies heavily on chemical control despite advances in microbiome-informed practices, resistant cultivars, and biotech traits^27^. Table 1 summarizes major pathogens, their impacts, and regions affected by notable outbreaks.

**Table 1:** Global Significance of Major Fungal Pathogens Affecting Agricultural Sectors

Sector	Fungal Disease	Pathogen	Host Crop	Impact	Region	Notes	References
Cereals	Fusarium Head Blight	*Fusarium graminearum*	Wheat, barley, maize	Up to 80% yield loss in outbreaks; DON contamination and grain downgrading	America, Europe, Asia	Food safety risk; DON (vomitoxin)	^176–178^
	Wheat Stem Rust	*Puccinia graminis f. sp. tritici*	Wheat	>10% yield losses; catastrophic in susceptible fields ( > 50% losses)	Eastern Africa, Middle East	Ug99 lineage undermines resistance	^179,180^
	Septoria Leaf Blotch	*Zymoseptoria tritici*	Wheat	Up to 50% yield losses in epidemics; billions in fungicide costs	Europe; temperate wheat-growing regions worldwide	Rapid azole resistance evolution	^181,182^
	Rice Blast	*Pyricularia oryzae*	Rice	Up to 30% yield losses globally	Asia, Africa, South America	Most destructive rice disease globally	^183^
	Aspergillus Ear Rot	*Aspergillus flavus*	Maize, groundnuts	Aflatoxin contamination; severe food safety risk	Africa, Asia, U.S.	Immunotoxic, carcinogenic mycotoxins	^184,185^
Legumes & Oilseeds	Soybean Rust	*Phakopsora pachyrhizi*	Soybeans	80-90% yield loss without control; $2B annual loss in Brazil	South America, Asia, Africa	High moisture promotes spread	^186,187^
	Sclerotinia Stem Rot (White Mold)	*Sclerotinia sclerotiorum*	Soybeans, Canola	$4.3B in U.S. losses (1996-2016); up to 50% yield loss	US, Canada	Favoured by cool, moist conditions	^188–190^
	Fusarium Wilt	*Fusarium oxysporum f. sp. ciceris*	Chickpeas	Up to 94% yield loss; early infections can cause complete crop failure	India, Australia, Mediterranean	Soil-borne; persists for years	^191,192^
Horticultural Crops	Early Blight	*Alternaria solani*	Tomatoes, Potatoes	Premature senescence and major yield losses (up to 79%); fruit-quality losses in severe epidemics	Worldwide	Humidity- and moisture-dependent	^193^
	Black Sigatoka	*Pseudocercospora fijiensis*	Bananas	Up to 50% yield loss	Tropics, Subtropics	Devastating in monoculture plantations	^194^
	Fusarium Wilt	*Fusarium oxysporum f. sp. Cubense* TR4	Bananas	No fungicidal control; threatens Cavendish monocultures	Worldwide	Long persistence in soil	^195,196^
	Gray Mold	*Botrytis cinerea*	Grapes, Strawberries	10-25% yield loss, >50% in outbreaks	Worldwide	Humidity-related post-harvest rot	^197,198^
	Apple Scab	*Venturia inaequalis*	Apples	Up to 70% losses in Russia; heavy fungicide use	Temperate regions	Most significant apple disease	^199,200^
	Black Spot	*Diplocarpon rosae*	Roses	Reduces market value; $203M to $168M loss in U.S.	Worldwide	Major esthetic/economic concern	^201,202^
	Root and Crown Rot	*Calonectria* spp.	Ornamentals (e.g., Boxwood)	Widespread plant loss in 30 + U.S. states	Europe, USA	Hard to eradicate; long-lived spores	^203,204^
Trees / forest species	Dutch Elm Disease	*Ophiostoma novo-ulmi*	Elm	Near-complete elm mortality across two continents	Europe, North America	Beetle-vectored; extremely hard to control	^205,206^
	Ash Dieback	*Hymenoscyphus fraxineus*	Ash (*Fraxinus excelsior*)	~85% mortality; £22B cost in UK	Europe	Major ecological and economic costs	^207,208^
	Chestnut Blight	*Cryphonectria parasitica*	Chestnut	~4 billion trees lost; severe ongoing decline in European chestnut.	North America, Europe, Asia	Highly destructive; restoration difficult	^209,210^
	Root Rot	*Armillaria* spp.	Fruit, Nut, Coniferous Trees	Millions $ annual losses; >200,000 ha affected in Poland	US, Poland, Australia	Difficult to detect/manage	^211–213^

This table highlights representative fungal diseases with substantial implications for food security, agricultural economies, and trade. Beyond individual crop losses, these pathogens collectively threaten oilseed and legume protein supplies, staple fruit and vegetable markets, forest health, and the global floriculture industry. Their persistence in soil, adaptability across climates, and the limited availability of resistant cultivars complicate management. Climate change, intensive monoculture systems, and high fungicide dependency influence disease spread and resistance evolution. Disclaimer: Regions listed indicate where the pathogen has been most frequently reported or caused major outbreaks; however, many outbreaks are globally distributed. Yield loss and economic impact vary with local climate, cultivar susceptibility, and management intensity.

Many plant-pathogenic fungi deploy mycotoxins as virulence factors that induce host cell death and facilitate infection^28^. Consequently, toxin-producers—particularly *Fusarium, Penicillium*, and *Aspergillus* species—sit at the nexus of food safety, public health, and antifungal resistance, with global surveys reporting detectable mycotoxins in the majority of cereal samples ( ≈ 60–80%) and EU- or Codex-based regulatory levels exceeded in approximately one-fifth of raw, non-prescreened grains, especially maize and wheat^29^. Warming and humidity further amplify contamination and biosynthesis of and contamination with deoxynivalenol (DON), fumonisins, and aflatoxins, while climate-driven range shifts are linked to emerging, under-monitored toxins (e.g., fusaproliferin, moniliformin)^30^.

Control versus therapeutic options diverge sharply between agriculture and medicine: more than 100 crop fungicides are available, but only six systemic classes are approved for the treatment of invasive fungal infections in humans^1,31^. Toxicological paradigms and use patterns are not transferable between these domains, compounds acceptable for chronic, low-dose environmental exposure may be unsafe for short-course, high-dose systemic therapy. For example, strobilurins [FRAC 11, QoIs] inhibit mitochondrial Complex III in both fungi and humans, creating unacceptable off-target risks, such as oxidative stress, mitochondrial injury, and organ toxicity. As a result, most crop fungicides are unsuitable for human use. Azoles/DMIs are the notable cross-domain exception, although their deployment demands careful stewardship^32,33^.

### Animal-pathogenic Fungi – pets, livestock, and wildlife

#### Pets

*Microsporum canis*, the major cause of zoonotic dermatophytosis in cats, is an important cause of dermatophytosis in humans^34^. Feline sporotrichosis (causative agents: *Sporothrix schenckii* and *Sporothrix brasiliensis)* has led to epidemic outbreaks and represents a significant zoonotic concern^35^. Environmental exposure of companion animals to opportunistic molds, such as *A. fumigatus* further illustrates the shared ecological niches linking animals, humans, and the environment^36^. Given increasing human interaction with pets, the number and severity of zoonotic infections are expected to increase.

#### Livestock

Large mammals, such as horses face important fungal threats shaped by environmental exposure, husbandry practices, and immune status, with dermatophytosis, cryptococcosis, and aspergillosis representing the most prevalent and globally distributed concerns. Equine dermatophytosis, although often self-limiting, typically warrants antifungal treatment because of its high contagion potential, zoonotic risk, and associated management considerations^37,38^.

In cattle, *Trichophyton (T.) verrucosum* remains a highly contagious agent of bovine dermatophytosis that can spread to farm workers. Recent isolates now include fluconazole-resistant *T. verrucosum*, raising concerns about emerging antifungal resistance in bovine dermatophytosis^39^. Mastitis in dairy herds is increasingly complicated by opportunistic eukaryotic pathogens, most notably *Prototheca bovis* and diverse *Candida* species. *P. bovis*, an achlorophyllous alga, is intrinsically resistant to many antimicrobial agents, making infections difficult to eradicate and contributing to chronic, recurrent mastitis^40^. In parallel, mycotic mastitis caused by *Candida* spp., particularly non-albicans species, such as *C. krusei* and *C. parapsilosis* are an important cause of disease and economic loss in dairy herds: in a Chinese survey, these species represented the dominant *Candida* isolates from clinical mastitis, showed high rates of resistance to azole antifungals and fluorocytosine^41^, while *Cryptococcus* (*Cr*.) *neoformans* has also been isolated from the mammary gland in some cases, indicating that cryptococcal infections, although rare, can involve the udder in dairy animals^42^.

#### Poultry

Intensive poultry production, high stocking densities, poor ventilation, and the accumulation of organic matter create environments that favour fungal growth and suppress immune function, including antibody- and cell-mediated responses^43^. *A. fumigatus* is the principal agent of avian aspergillosis, thriving in contaminated litter and poorly ventilated housing, where inhalation of airborne spores from organic-rich litter leads to granulomatous lesions of the lungs and air sacs, dyspnea, and high mortality, particularly in young birds^44^. Opportunistic fungal infections caused by *C. albicans* have also been documented in stressed or immunocompromised birds^45^. Because poultry farms maintain tightly controlled microenvironments, recurrent azole use in bedding, hatcheries, and housing facilities intersects with broader environmental reservoirs, positioning poultry houses as interfaces for the selection and dissemination of azole-resistant *A. fumigatus* within poultry production systems^46^.

#### Aquacultures

Fungal and fungal-like pathogens present an increasing concern in aquaculture, where dense stocking conditions, ecological stress, and poor water quality promote infections. *Saprolegnia* species, a genus of oomycetes, represent particularly troublesome pathogens in salmon aquaculture, infecting both the skin and gills and impairing osmoregulation and respiration, leading to increased mortality in young fish and significant losses in hatcheries and commercial farms. Although azoles are not the primary therapeutic intervention for *Saprolegnia* infections, secondary fungal infections, such as *Exophiala* spp. occasionally necessitate their use. In marine aquaculture, however, azole treatments have shown limited efficacy, as infections in marine fishes including elasmobranchs are frequently caused by intrinsically azole-resistant *Fusarium* and *Neocosmospora* species. Concern about azole use in aquaculture is therefore growing due to their limited efficacy against several aquatic fungal pathogens and the potential for resistance development^47–49^.

#### Wildlife

**Fig. 2:**
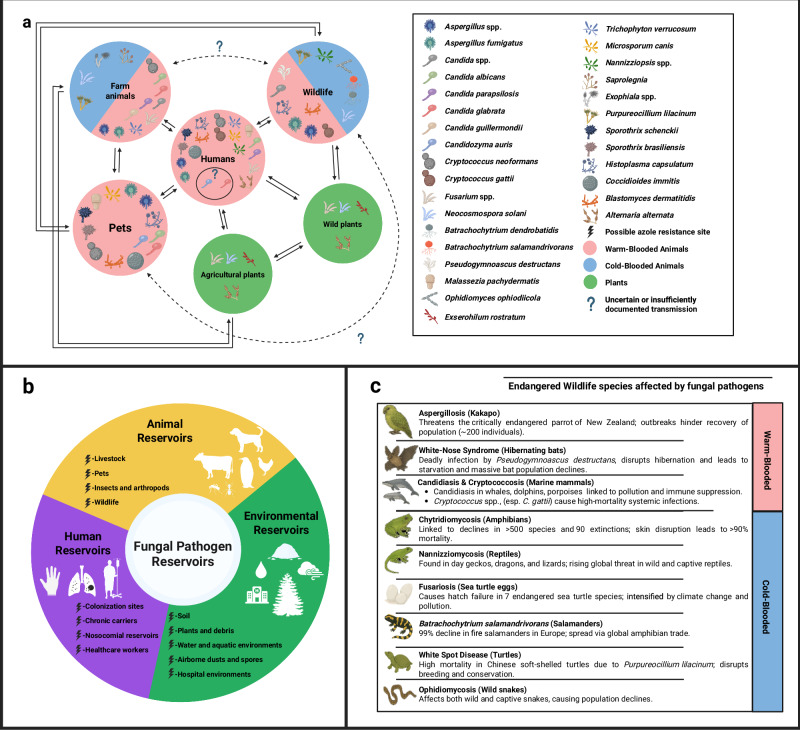
Cross-Sector Transmission Pathways (a), One Health Reservoirs (b), and Wildlife Threats (c) of Fungal Pathogens. **a** Transmission interface diagram illustrating key pathogenic fungal species of importance in animal–human transmission. Warm-blooded animal reservoirs are the main focus for pets, while acknowledging that cold-blooded pets (e.g., fish, reptiles, amphibians) can also act as carriers. Arrows indicate documented or plausible transmission routes between humans, domestic animals, wildlife, and plants. **b** Major fungal pathogen reservoirs within the One Health framework, spanning human, animal, and environmental domains. **c** Examples of wildlife species affected by pathogenic fungi, including both warm-blooded and cold-blooded hosts, with conservation-relevant outcomes (e.g., mass mortality, population decline). Representative examples of cross-species transmission, One Health reservoirs, and wildlife impacts are supported by refs. ^51,98,231–252^. Created in BioRender. ROOHI, B. (2026) https://BioRender.com/a2tjx4s. Disclaimer: This schematic is not exhaustive. It highlights representative pathogenic species and key pathways to illustrate potential cross-species transmission risks rather than providing a complete epidemiological mapping.

Protecting endangered wildlife is critical for maintaining biodiversity and ecosystem balance, yet emerging fungal diseases are increasingly driving population declines across diverse taxa. Fungi play diverse ecological roles, including decomposition and symbiosis, but they can also act as major pathogens that drive population declines and biodiversity loss. They have been implicated in approximately 72% of pathogen-driven extinction or extirpation events and considered responsible for ~65% of pathogen-driven host loss in plants and animals. Human activities, including habitat loss, international trade, and climate change, have accelerated the emergence and spread of invasive fungal pathogens, intensifying disease pressure in vulnerable wildlife, such as bats, amphibians, corals, and turtles and contributing to ongoing biodiversity loss^15,50^. In conservation settings, antifungal interventions, often reliant on azole-based treatments with limited effectiveness, are among the few available tools to manage infections in species lacking natural immunity or facing newly emerged pathogens, although their effectiveness remains limited^15,50,51^. Figure 2a–c integrates these themes by mapping major reservoirs, illustrating representative endangered wildlife cases across warm- and cold-blooded hosts, and outlining interfaces among humans, pets, livestock, wildlife, plant hosts, and environmental sources.

### Human-pathogenic fungi

#### Dermatomycoses and other superficical mycoses

Superficial fungal infections, dominated by dermatomycoses, affect around 20–25% of the global population, making them among the most common human infections. Tinea corporis and Tinea pedis are most often caused by *T. rubrum* and *T. interdigitale*, while Tinea capitis frequently involves zoophilic agents, such as *M. canis* or anthropophilic *T. tonsurans*, depending on the region. Zoonotic transmission from domestic animals and indirect spread via contaminated surfaces in communal environments, such as swimming pools and locker rooms facilitate dissemination. Azoles and terbinafine remain first-line therapies, but antifungal resistance is increasingly reported in several *Trichophyton* species, especially in patients with recurrent disease^52–54^. Superficial mycoses affect keratinized tissues, such as skin, hair, and nails, causing infections like onychomycosis and otomycosis^55^. In the U.S., onychomycosis affects approximately 10–14% of adults, predominantly involving the toenails, commonly caused by *T. rubrum* and *T. mentagrophytes*^56^. A new problem is the emergence of a highly virulent lineage within the *T. mentagrophytes/interdigitale* complex, now referred to as *T. indotineae*. It shows high degrees of resistance to two currently used antifungal classes, i.e., terbinafine and azoles. Resistance has possibly been enhanced by inappropriate over-the-counter use of antifungal agents^57^. Nail infections caused by *Fusarium* species, though less common, are concerning due to their potential for systemic dissemination in immunocompromised patients^58^.

#### Subcutaneous mycoses

Subcutaneous fungal infections penetrate deep into dermal and soft tissues, often due to trauma (e.g., cat scratches) or occupational exposure, and are common among farmers and outdoor laborers in endemic regions^58^. Key infections include sporotrichosis and chromoblastomycosis caused by *S. schenckii* and *Fonsecaea pedrosoi*, respectively^59,60^. These chronic infections often require prolonged antifungal therapy, typically with itraconazole. However, long treatment durations pose clinical challenges, including variable treatment response in advanced or fibrotic lesions and the need for extended therapy to prevent relapse^61^. Sporadic azole resistance has been reported in environmental fungal pathogens in regions with high agricultural fungicide exposure, complicating treatment^62,63^.

#### Disseminated (systemic) mycoses

Systemic fungal infections are life-threatening, particularly in immunocompromised individuals. Opportunistic fungi, such as *Candida* spp., *A. fumigatus*, and *Cr. neoformans*, as well as geographically restricted dimorphic fungi like *Histoplasma capsulatum* and *Coccidioides* (*Co*.) *immitis*, are among the most prevalent pathogens in this category^17^. Systemic candidiasis is a leading cause of nosocomial infections, with mortality rates of 40–55%, while invasive aspergillosis has mortality rates ranging from 43 to 72% in COPD patients and can approach 90% in critically ill ICU patients with severe underlying conditions^17,64^.

#### Cross-domain opportunistic fungi

**Fig. 3:**
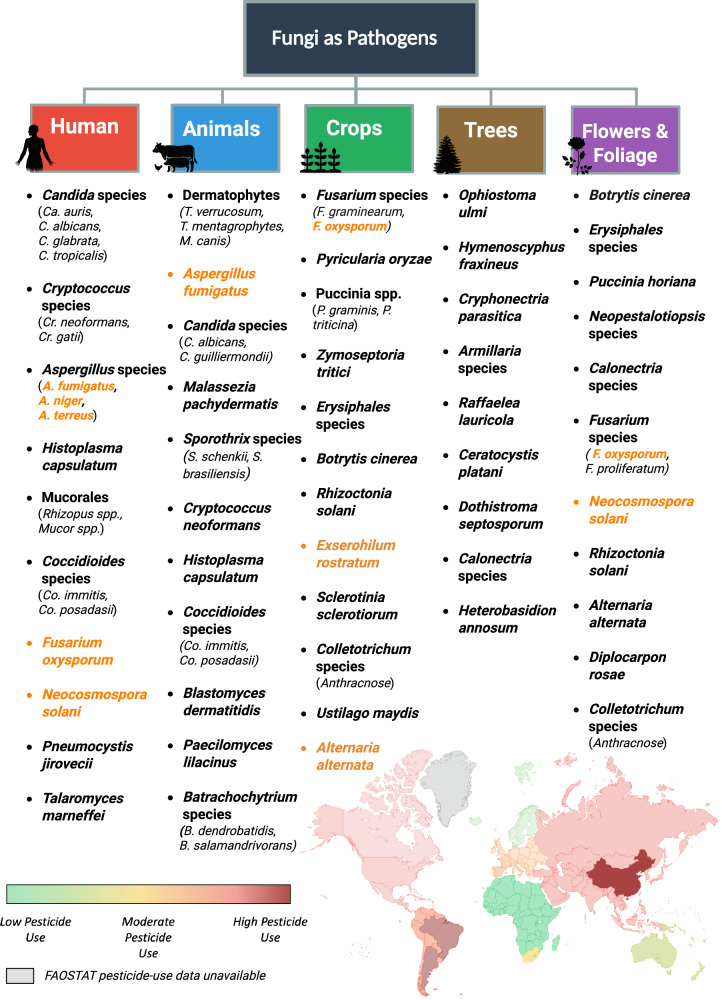
Major fungal pathogens affecting humans, animals, crops, trees, and flowers, including cross-domain opportunists. Cross-domain opportunists, highlighted in orange, infect multiple host types, reinforcing the need for the One Health framework. Species selection is based on documented prevalence, economic significance, and cross-sector impact in global reviews of human, veterinary, plant, and environmental mycoses^23,50,98,253–262^. Background shading (heatmap): relative intensity of overall fungicide use, shown as a gradient from low to high fungicide input. Values are based on FAOSTAT “fungicides and bactericides” use per hectare of harvested cropland for 2013–2022, aggregated by continent^263^. As FAOSTAT does not distinguish active ingredients, this shading reflects total fungicide inputs rather than azole-specific use, and does not capture differences in fungicide composition (e.g., multisite [FRAC M] vs azole products). The map is therefore intended as contextual background for plant-health pressure, *not* as a quantitative estimate of azole selection pressure. Comparable global, harmonized datasets describing antifungal consumption across human and veterinary sectors are not currently available in spatially comparable formats to FAOSTAT pesticide-use statistics. Regional differences in healthcare access, diagnostics, crop-loss reporting, and biodiversity monitoring mean that the true fungal burden is likely underestimated, particularly in low-resource settings^50,263,264^. Created in BioRender. ROOHI, B. (2026) https://BioRender.com/fpn2mm9.

Beyond classical zoonotic and environmentally acquired fungi, some fungal species exhibit notable ecological versatility, persisting across plants, animals, humans, and environmental niches. Traditionally, fungal pathogens have been classified by taxonomy, ecological roles, or disease spectrum. However, a number of ecologically versatile fungi appear to challenge this framework by persisting in diverse reservoirs and occasionally infecting biologically distinct hosts. We propose the term “cross-domain opportunists” to describe this group, which extends the One Health framework by highlighting ecological overlap and host adaptability while complementing rather than replacing established categories of zoonotic or sapronotic transmission. The concept of cross-domain opportunists does not suggest primary adaptation to multiple hosts, but instead emphasizes opportunistic colonization and persistence across ecologically and biologically distinct environments, consistent with contemporary views of opportunistic pathogens. Notable examples of such fungi include *Fusarium oxysporum*, *Neocosmospora solani* (formerly *Fusarium solani*) and *Aspergillus* species (*A. fumigatus, A. niger, A. terreus*), which are known to affect humans, animals, and plants^65,66^. These species demonstrate remarkable ecological flexibility, thriving in diverse habitats while retaining the ability to impact multiple host types. Beyond these recognized pathogens, other fungal taxa exhibit significant host adaptability. For instance, *Candidozyma* (*Ca*.) *auris* (formerly *Candida auris)* and *Cr. neoformans* persist in non-host environments, such as soil and water, maintaining viability and transmission potential. Similarly, *Sporothrix* species (*S. schenckii, S. brasiliensis*) are found in environmental reservoirs and zoonotic infections^67–69^. In Brazil, *Sporothrix brasiliensis* infections show seasonal trends, peaking during cooler, drier months. Feline transmission remains a key factor in outbreaks, but seasonality suggests ecological and climatic triggers also play a role^70^. Fungi, such as *Exserohilum rostratum* are major agricultural pathogens, mainly on grasses, such as rice and wheat but also on other crops. Numerous cases of human infection have been reported, varying from skin and eye infections to disseminated disease^71^. Of particular significance was an outbreak of meningitis due to contaminated methylprednisolone injection, which led to 55 deaths^72^. Like *Fusarium* and *Neocosmospora*, this fungus has been associated with cross-domain pathogenicity, causing extensive crop damage while highlighting the potential for cross-domain interactions across multiple environmental niches^73^. It can be expected that additional fungal species with cross-domain infectious potential will be identified, emphasizing the need to explore their ecological roles and evolutionary mechanisms (Fig. 3)^74^.

#### Fungal pathogens of One Health importance

In addition to cross-domain opportunists, well-established zoonotic and environmentally acquired pathogens illustrate the One Health impact of shared environments between humans and animals. These organisms, though not persisting across all host groups, pose increasing challenges as ecological and epidemiological factors drive their prevalence^74^. Examples include histoplasmosis and coccidioidomycosis, respectively caused by *H. capsulatum* and *Co. immitis*^75,76^. Histoplasmosis typically arises from exposure to bat or bird droppings, where inhalation of fungal spores leads to pulmonary disease that may progress to disseminated infection in immunocompromised individuals, in whom mortality rates are substantially higher^76^. Similarly, coccidioidomycosis (“Valley Fever”), caused by *Co. immitis*, is linked to soil disturbance in endemic regions, with inhaled spores producing outcomes that range from mild respiratory illness to severe pneumonia and systemic dissemination in both humans and animals^75,77^. Some members of the Mucorales (*Mucor* and *Rhizopus* species) also display host plasticity in adapting to diverse ecological and biological conditions^78^. Climate change is further reshaping the distribution and impact of environmentally associated fungi. Warming climates are projected to expand the geographic range of *Co. immitis* in the United States by nearly 50% by the end of the century, while extreme weather events facilitate long-distance dispersal of pathogens, such as *Apophysomyces trapeziformis* and *Talaromyces marneffei*^79–81^.

Emerging fungal pathogens of potential One Health concern include antifungal-resistant *C. tropicalis*, detected in marine environments, such as aquarium dolphins and wild cetaceans. Enhanced survivability in high-salinity conditions raises concern about environmental barriers and risk of potential spillover into human health settings^82,83^. Another concern is *Blastomyces dermatitidis*, the cause of blastomycosis which affects the respiratory system and potentially spreads systemically. It infects dogs and humans, and occasionally other mammals, with pulmonary symptoms often progressing to severe disseminated infections in individuals with pre-existing lung conditions^84^. *Cr. neoformans* remains a notable environmentally acquired fungal pathogen of One Health relevance, with pigeons, bats, and pets serving as environmental reservoirs. Inhalation of aerosolized spores, particularly from contaminated droppings in urban areas, poses a significant risk to immunocompromised individuals, often leading to life-threatening cryptococcal meningoencephalitis (see Fig. 2a)^85,86^.

### Role of azoles across sectors

#### DMIs in agriculture

Globally, reliance on fungicides has increased sharply. Total pesticide sales, including fungicides, have doubled since 1990 and now exceed 3.7 million tonnes annually. Market projections indicate further increases driven by climate change, intensifying disease pressure on crops, and expanding agricultural production^87,88^.

Azoles are among the most widely used fungicides in agriculture, valued for their effectiveness against phytopathogenic fungi. Compounds like tebuconazole [FRAC 3, DMI], prothioconazole [FRAC 3, DMI], and difenoconazole [FRAC 3, DMI] are extensively used to protect staple crops, such as cereals (particularly wheat), as well as, selected horticultural crops including cucumbers and citrus, from fungal infections caused by genera like *Fusarium* and *Penicillium*^89^. A recent meta-analysis quantified the effects of triazole-based fungicides on wheat yield and test weight under FHB pressure, demonstrating their agronomic importance^90^. However, intensive azole use has been associated with the emergence of azole resistance mechanisms in filamentous fungi, including alterations in CYP51 genes and increased efflux pump activity^89,91^. Alarmingly, agriculture-driven resistance mechanisms have been linked to the emergence of antifungal-resistant infections in clinical settings, particularly for environmentally acquired fungal pathogens^92^.

In contrast to many multisite contact fungicides, azoles inhibit fungal sterol biosynthesis by targeting CYP51. This disruption compromises fungal cell membrane integrity, leading to increased permeability and impaired cellular processes. Azoles stand out from fungicide classes like strobilurins because they act systemically within plants, protecting both treated and new tissues from infection^93^. Their ability to safeguard high-value crops, such as cereals, fruits, and vegetables, together with their broad efficacy and systemic activity, has made azoles central to modern crop protection strategies^94^.

At the same time, increasing awareness of the ecological consequences of intensive azole use has stimulated interest in alternative disease-control strategies. In agricultural systems, synthetic azoles, such as tebuconazole have been reported to alter phyllosphere microbial community composition, with some studies suggesting potential consequences for microbial diversity and ecosystem resilience. By contrast, alternative approaches, including elemental sulfur [FRAC M2] and biological control agents, tend to preserve microbial diversity and are often considered more compatible with long-term sustainability goals^95^. Copper-based fungicides, which have been used since the eighteenth century, are still widely applied, particularly in viticulture. However, their repeated use leads to copper accumulation in soils, prompting regulatory and agronomic efforts to reduce inputs. Microbial biofungicides offer residue-free and biodegradable options, but their practical utility is often limited by low persistence, sensitivity to environmental conditions, and reduced efficacy under high disease pressure. Some products, such as the hyperparasite *Ampelomyces quisqualis*, are effective only within narrow temperature and humidity ranges, constraining their broader deployment^96^.

#### Azoles in veterinary medicine

Veterinarians rely on azoles for dealing with diverse fungal conditions of canines and felines, such as dermatophytosis, otitis externa, and systemic mycoses. In dogs and cats, the common commensal yeast *Malassezia pachydermatis* associated with seborrheic dermatitis and otitis externa, is frequently treated with topical azoles, such as miconazole or ketoconazole. Zoonotic transmission has been documented, underscoring the importance of hygiene practices, particularly for immunocompromised individuals^97,98^.

Feline dermatophytosis is commonly managed with systemic antifungals, such as itraconazole. However, the emergence of multi-azole-resistant *M. canis* strains has been documented, with cases showing recurrence and therapeutic failure following itraconazole therapy^99^. In parallel, environmental azole exposure is known to select for resistant fungal pathogens, such as *A. fumigatus*, and resistance in animal-associated isolates can compromise antifungal efficacy in veterinary practice^100^.

Azoles, such as itraconazole and fluconazole are commonly prescribed to manage fungal infections in animals, including pets, livestock and wildlife, due to their efficacy across phylogenetically diverse fungi. However, surveillance of antifungal resistance in animals remains limited, and available reports already document azole resistance in animal-associated fungal pathogens, highlighting the role of animals in the emergence of azole resistance^98^.

In veterinary medicine, azole exposure drives the selection and spread of resistant strains. Poultry farms act as hotspots for azole-resistant *Aspergillus* strains. Resistant airborne conidia originating from these environments can disperse over long distances, contributing to environmental spread and human acquisition of azole-resistant infections^36,101–103^. In poultry, combined use of azoles for fungal treatment and environmental exposure further accelerates resistance^104,105^.

Certain VMPs may also increase the risk of resistance in zoonotic fungi like *T. mentagrophytes* and *M. canis*, which affect both animals and humans. Cross-species transmission is particularly concerning in shared human-animal environments^106,107^. In *T. mentagrophytes*, azole resistance arises from CYP51B amplification, while squalene epoxidase gene mutations contribute to multidrug resistance in *T. indotineae*^108,109^.

Wildlife is also at risk, with invasive fungal infections (including aspergillosis) reported in avian scavengers from agricultural and human-impacted environments^110^. Birds, due to their high body temperatures and migratory behaviour, may act as reservoirs for thermotolerant fungi like *Ca. auris*, potentially contributing to its global spread across One Health interfaces^111,112^.

#### Azoles in human medicine

**Fig. 4:**
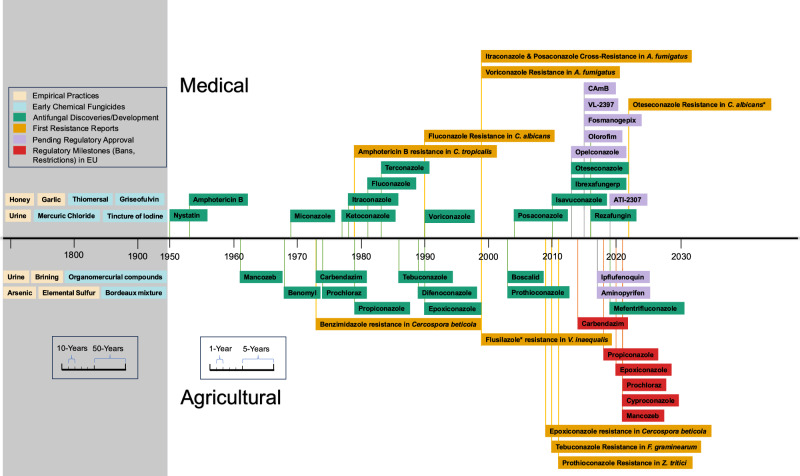
Evolution of medical and agricultural antifungal compounds, emergence of resistance, and regulatory responses. Increased MICs observed in vitro, linked to upregulation of efflux pumps (CDR1, MDR1) and target enzyme (CYP51); clinical significance remains undetermined. Source: FDA VIVJOA Label, 2022. This timeline outlines key milestones around antifungal drug development from early treatments to synthetic modern compounds. It features antifungals used clinically and agriculturally and fungal pathogens resistant to azoles that emerged. It also highlights regulatory actions in limiting antifungal use. Successive generations of azoles advanced greatly in both safety and selectivity through improved targeting of fungal CYP51 enzymes, from early topical imidazoles like clotrimazole and miconazole to oral ketoconazole in the 1970s, and itraconazole in the 1980s, as well as later creating the better tolerated triazoles, such as fluconazole, voriconazole, posaconazole and isavuconazole. Recent innovations, such as the tetrazoles oetseconazole and VT-1598 seek to improve fungal specificity while reducing off-target toxicity. Agricultural fungicides displayed, show the parallel historical development of PPPs. Benzimidazoles (MBCs) [FRAC 1], Amphotericin B (Poleyenes) and other non-azole classes are included solely as historical markers within the broader development of agricultural fungicides; their presence does not imply cross-resistance with medical azoles. Regulatory restrictions on several PPPs (e.g., propiconazole, carbendazim [FRAC 1]) in the EU, have been primarily driven by toxicological and endocrine-disruption assessments. These milestones are shown in the timeline solely to contextualize the regulatory evolution of fungicide use, and are not linked to clinical antifungal resistance. Historical and mechanistic references supporting antifungal classes, resistance events, and regulatory milestones are provided in refs. ^265–275^ and Supplementary Table 2. Created in Microsoft PowerPoint 365. Disclaimer: Only resistance events that (i) are supported by primary, peer-reviewed evidence, (ii) involve economically or ecologically important plant pathogens, and (iii) serve as representative milestones in documented resistance evolution were included. These examples illustrate the chronological emergence of major resistance phenotypes but are not intended as an exhaustive list.

Azoles reshaped antifungal therapy by providing orally available and less toxic options at a time when systemic treatment depended largely on amphotericin B deoxycholate and flucytosine. Amphotericin B deoxycholate was effective but highly nephrotoxic, while flucytosine, an anticancer derivative, was associated with bone marrow toxicity, including leukopenia and aenemia, limiting the clinical utility of both agents. The arrival of the triazoles expanded therapeutic possibilities. Fluconazole offered a well-tolerated option for major yeast infections, while itraconazole and voriconazole improved the management of invasive fungal diseases, including invasive aspergillosis. Later agents, such as posaconazole and isavuconazole further broadened the spectrum of activity and consolidated the role of azoles in both prevention and treatment of invasive yeast and mold infections. Unlike fungicidal agents, such as amphotericin B, most medical azoles are fungistatic, particularly against yeasts. This suppressive mode of action may permit the survival of tolerant fungal subpopulations and potentially contribute to the emergence of antifungal resistance. More recently, development of the tetrazole subclass successfully focused on improving fungal selectivity and minimizing host toxicity, with the approval of Oteseconazole for recurrent vulvovaginal candidiasis, exemplifying these advances (Fig. 4, Supplementary Table 2)^113^.

In clinical practice, azoles are deployed across the full spectrum of fungal disease, from superficial and mucocutaneous infections to invasive mycoses requiring long-term treatment or prophylaxis^114^. Topical azoles, such as clotrimazole and miconazole are widely used, while systemic therapy is required for severe or refractory cases. Ketoconazole and imidazole derivatives are widely used in cosmetic and hygiene products for their antifungal properties. Ketoconazole-based shampoos effectively treat *Malassezia* infections, the primary cause of dandruff and seborrheic dermatitis, while imidazole-containing creams and powders are standard for *Trichophyton* infections like athlete’s foot^115–117^. In high-risk settings, including organ transplantation and haematologic malignancy, systemic triazoles, such as fluconazole, voriconazole, posaconazole, and isavuconazole remain central to antifungal prophylaxis and treatment strategies^118–120^. Voriconazole’s superior central nervous system (CNS) penetration makes it particularly effective for neuroaspergillosis, where fungal invasion of the CNS presents significant therapeutic challenges^121,122^.

Azoles are generally safe and well-tolerated, but systemic and long-term use can lead to significant side effects, particularly in specific patient populations. Hepatotoxicity is one of the most frequent adverse effects with triazoles, such as voriconazole, and routine liver function monitoring is therefore recommended during prolonged therapy^123^. Azoles inhibit cytochrome P450 enzymes, leading to significant drug–drug interactions, especially with immunosuppressants, such as tacrolimus and cyclosporine. As a result, therapeutic drug monitoring is essential in transplant recipients and other high-risk groups^124^. Ketoconazole, a synthetic imidazole antifungal, inhibits multiple cytochrome P450 enzymes involved in adrenal steroidogenesis and lowers both cortisol and androgen levels. Because of these endocrine effects, it is widely used off-label as a medical therapy for Cushing’s syndrome^125^. Voriconazole has been linked to neurotoxic effects, including visual hallucinations, confusion, and encephalopathy, particularly at high plasma concentrations^122^. It also causes photosensitivity and phototoxic skin reactions, which can progress to skin malignancies in chronically treated patients, especially transplant recipients^126^. Fluconazole, on the other hand, may prolong QT intervals (ventricular depolarization–repolarization time) on the electrocardiogram, especially in patients with additional risk factors or concomitant medications^127^.

These side effects are of greater concern when triazoles are used for prophylaxis or treatment of invasive fungal infections, where toxicities can occur with excessive systemic drug exposures, reinforcing the need for careful dosing and monitoring in transplant medicine and haemato-oncology^128^.

### Costs of the dual use of azoles: azole pollution and azole resistance

#### Azole pollution

The extensive use of azoles in agriculture has led to widespread environmental contamination, particularly through agricultural runoff into aquatic ecosystems. Sub-lethal azole concentrations in these environments create a “reservoir effect,” selecting for resistant fungi and potentially enabling horizontal transfer of resistance determinants to medically significant pathogens^129–131^. Azole residues from both personal-care products (e.g., fluconazole, climbazole) and agricultural fungicides (e.g., imazalil [FRAC 3, DMI], tebuconazole) have been detected together in surface waters and wastewater at significant concentrations (frequently exceeding 100 ng/L, with fluconazole reaching up to 739 ng/L in effluents and tebuconazole up to 1.7 µg/L in surface waters), demonstrating their co-occurrence and incomplete removal with sustained presence in aquatic environments. Although these studies demonstrate widespread environmental occurrence, reported concentrations are generally interpreted in relation to toxicological thresholds, and concerns primarily relate to chronic exposure and resistance selection rather than acute toxicity^132^. Agricultural use remains the dominant contributor to environmental azole loads, with fungicides detected in more than 75% of monitoring samples from agricultural catchments including streams, drainage canals, and field-adjacent sediments with certain regions reaching detection frequencies of up to 96%^129^. Soil and sediment act as long-term sinks for azoles, reflecting repeated agricultural applications. In intensively managed agricultural regions, environmental azole levels exhibit clear seasonal variation associated with periods of increased fungicide use^133^. Diffusive contamination pathways include spray drift, stormwater runoff, and deposition from treated materials^8^. Unsurprisingly, elevated azole levels have been detected in urban gardens and forests near farmland through diffusive environmental transport, including aerial dispersion and surface transfer processes^134^.

Azole fungicides exhibit high environmental mobility and persistence, remaining detectable in soil, surface water, and, in some cases, groundwater used for drinking water. Compounds, such as propiconazole [FRAC 3, DMI] can persist in soil for over 315 days, while tebuconazole may remain for up to 263 days under certain conditions^135,136^. This persistence disrupts microbial ecosystems, contributes to the selection of resistant fungal populations, and poses direct toxicological risks, collectively exerting sustained selective pressure that accelerates resistance development with implications for human, animal, and ecosystem health^129,137,138^. Environmental degradation rates are influenced by factors including pH, temperature, microbial activity, and application intensity, contributing to marked spatial and temporal variability in azole persistence^139^.

In addition to agricultural and domestic sources, industrial and manufacturing effluents, as well as leaching from treated construction materials and antifouling or marine coatings, have been identified as potential contributors to environmental azole contamination; however, quantitative data on their relative contribution remain limited, these sources fall outside the primary scope of the present review^4^.

#### Azoles in food and feed

Azole fungicides are frequently detected in food commodities, reflecting their extensive use during crop production and post-harvest preservation. In a survey of 84 commercial red wines from the Canary Islands, the Iberian Peninsula and Cape Verde, 81% of samples contained at least one quantifiable pesticide residue, and in the subset of Cape Verde wines, the azole fungicide tebuconazole was detected in all samples^140^. Fruit and vegetables, including commonly consumed produce, such as cucumbers and tomatoes, can contain measurable azole fungicide residues. For example, field-treated samples have been reported to carry tebuconazole concentrations ranging from 3.5-14.6 µg/kg^141^.

Post-harvest azole treatments for fruits and vegetables contribute to sustained fungicide exposure and selection pressure in food-handling systems. Post-harvest treatment with fungicides like imazalil is widely used, especially on citrus to suppress fungal decay and extend storage, with demonstrated efficacy in preserving quality^142^. Its use warrants careful stewardship given evidence of androgen‑receptor antagonism and endocrine effects in rodent models following maternal exposure^143^. Subtherapeutic azole levels can alter gut microbiota by suppressing protective commensals and promoting dysbiosis^144^. Such dysbiotic states may, in principle, create ecological niches permissive to opportunistic fungi, including *Candida*, and could theoretically contribute to antifungal selection pressure under chronic exposure, although direct experimental or clinical evidence remains limited.

Furthermore, oral exposure to agricultural triazole fungicides has been shown in mammalian models to disrupt intestinal homeostasis. Epoxiconazole [FRAC 3, DMI] ingestion in mice alters gut microbiota composition, modifies mucus secretion, and impairs epithelial barrier function^145^. These findings provide biologically plausible pathways through which dietary fungicide residues might influence gut microbial ecology. However, direct evidence from human studies is currently lacking.

#### Impact of azoles on wildlife

Wildlife inhabiting agricultural landscapes accumulate azole fungicides through dietary and habitat-associated exposure. For instance, blackbirds (*Turdus merula*) in vineyards exhibit elevated levels of azoles like tetraconazole [FRAC 3, DMI] and tebuconazole, particularly during peak agricultural seasons, while birds in non-agricultural areas show minimal contamination^134^ In natural ecosystems, azole fungicides disrupt biodiversity. For instance, bioaccumulation of imidazoles like climbazole and clotrimazole in aquatic organisms impairs endocrine systems, alters sex hormone levels, and destabilizes aquatic food webs^146,147^.

#### Toxicology/exposure–effect interpretation for azole DMIs

Across recognized environmental “selection hotspots” for *A. fumigatus*, measured residues of agricultural azole DMIs (e.g., tebuconazole, propiconazole, epoxiconazole, difenoconazole) in plant waste, flower-bulb residues, and compost commonly reach the µg·kg⁻¹–mg·kg⁻¹ band, overlapping concentrations associated with selection of TR-mediated resistance during thermophilic composting and waste handling, even though these levels are far below mammalian systemic toxicity thresholds derived by EFSA/ECHA (European Chemicals Agency) for the same substances^130,148–150^. In agricultural waste after labeled use, typical post-application residues are in the tens to hundreds of µg·kg⁻¹ range for several DMIs, which are below clinical MICs for medical azoles, but plausibly within the sub-inhibitory window that can enrich resistant *A. fumigatus* genotypes under repeated exposures. The recent EFSA report provides a provisional approach to calculate the predicted no effect concentration for resistance selection (PNECres) in *A. fumigatus* using MIC determinations. Their risk assessment identified various potential hotspots for resistance selection (e.g. maize and wine grape production) where DMI residue levels exceeded PNECres^4^. many *A. fumigatus* isolates that possess TR-mediated azole resistance mutations may also carry additional mutations conferring resistance to other fungicide classes, such as QoI or MBC compounds, complicating resistance selection dynamics in agricultural waste. This might explain the weak and non-significant correlations that were observed between DMI levels and resistance frequency in flower bulb waste. Medical azoles (fluconazole, itraconazole, voriconazole) detected in wastewater and receiving waters typically occur at ng·L⁻¹–µg·L⁻¹. While these levels are well below therapeutic plasma concentrations and derived toxicological thresholds, they can fall within the minimal selection concentration (MSC) band for resistance selection in environmental biofilms and sediments given prolonged exposure and large population sizes (Supplementary Table 3)^151–153^.

### Azole resistance, azole resistance mechanisms, and prominent examples

#### Azole resistance in human-pathogenic fungi

Surveillance studies consistently show that a substantial share of azole-resistant *A. fumigatus* infections arise in patients without prior azole exposure, supporting an environmental origin of resistance. In a nationwide Dutch study, 64% of patients with an azole-resistant isolate were azole-naive at the time of sampling^154^, and global clinical data similarly indicate that two-thirds of patients with azole-resistant aspergillosis had no previous azole therapy. Resistance prevalence also varies across Europe. Dutch centers have reported 4–13% azole resistance among culture-positive patients^155^, while other countries generally report lower rates, reflecting differences in environmental exposure and fungicide selection pressures. This suggested that resistance selection occurred in the environment, with patients inhaling resistant *Aspergillus* conidia. Studies identified the TR_34_/L98H mutation in the Cyp51A gene in resistant isolates, which were also recovered from agricultural environments^156^. Triazole fungicides, first authorized in 1990, were linked to the emergence of azole-resistant *A. fumigatus* by 1998^157^. Resistance selection occurs in agricultural waste streams, where *A. fumigatus* thrives on decaying plant material and residues of DMI fungicides promote resistant clones^130^. A second resistance mutation, TR_46_/Y121F/T289A, was reported in 2009, and both mutations are now globally prevalent in environmental and clinical samples^158^. Voriconazole-resistant *A. fumigatus* is associated with a 25% higher mortality at day 90 compared to susceptible infections, negating the survival benefits of voriconazole therapy^159^. Recent studies reveal increasing genetic diversity in TR_34_ and TR_46_ backgrounds, as well as mixed-genotype infections, complicating diagnosis and treatment^160^. Beyond healthcare, resistant fungal spores in urban and agricultural areas exacerbate conditions like fungal asthma and complicate clinical management^92,161^. Environmental azole exposure is increasingly linked to resistance in other human fungal pathogens. For example, in *C. tropicalis* fluconazole resistance has been associated with exposure to agricultural azoles, such as difenoconazole, tebuconazole, and triadimenol [FRAC 3, DMI], with resistant isolates recovered from water, soil, and fruits in Taiwan^162^. Environmental and clinical resistant *C. tropicalis* isolates clustered within a single clade (Clade 4), indicating a shared lineage between environmental and human sources. A study in Brazil further demonstrated environmental resistance selection across multiple yeast species. Sampling from patients, hospital environments, poultry, pigs, and animal habitats revealed that 3.6% of *Candida* isolates were fluconazole-resistant and exhibited high MICs for tebuconazole, particularly in *C. albicans, C. tropicalis*, and *C. krusei*^163^. Furthermore, the global emergence of *Ca. auris* illustrates the clinical impact of azole resistance in large international collections. About 90–93% of isolates are fluconazole-resistant, and 15–35% show reduced susceptibility to amphotericin B, whereas only a smaller minority (around 2–8%) are resistant to echinocandins. Within this resistance profile, treatment options are limited and current guidelines recommend echinocandins as the preferred initial therapy for *Ca. auris* infections^164,165^.

### Common mechanisms of azole resistance in fungi

**Fig. 5:**
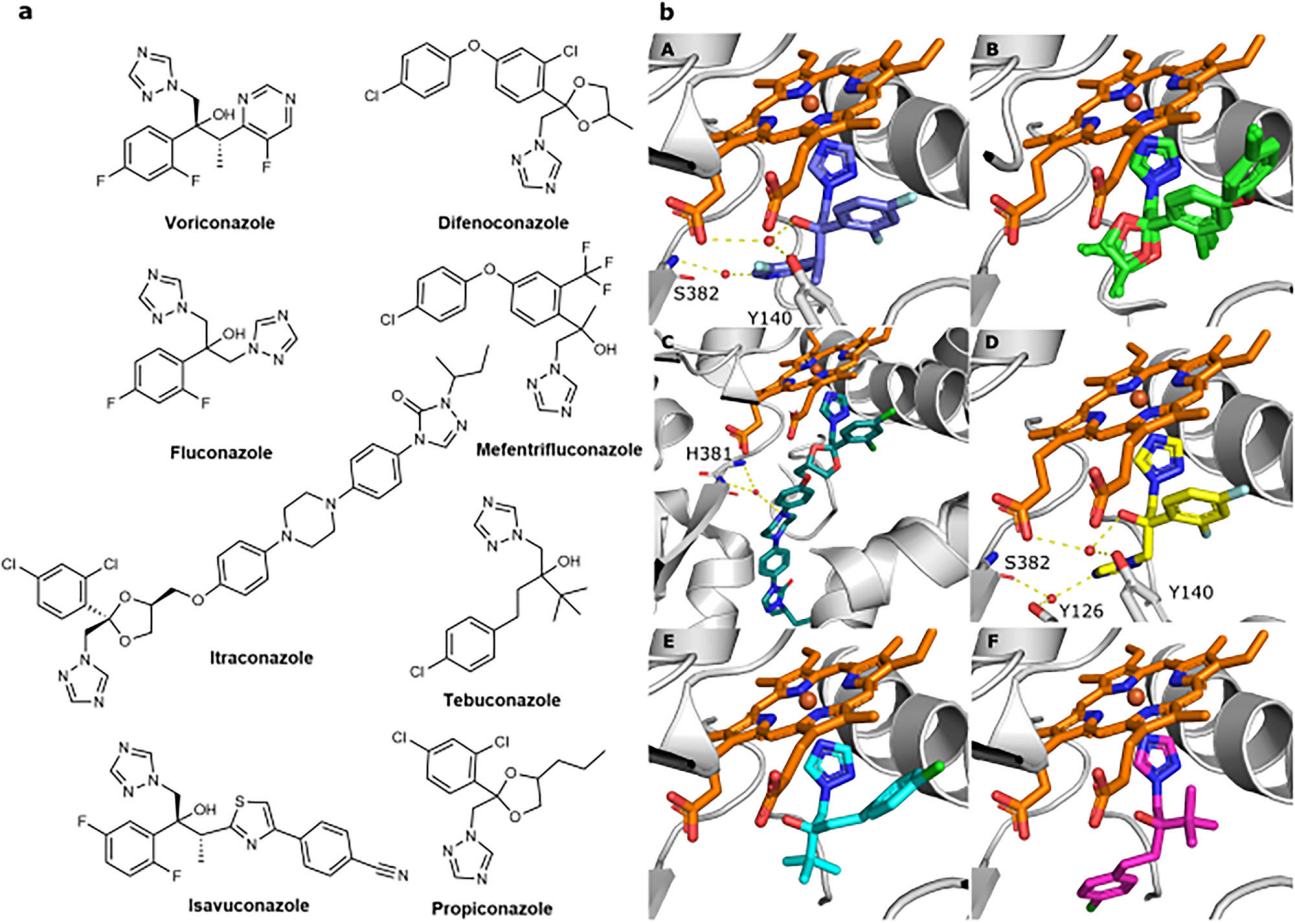
Structural Comparison (a) and Binding Interactions (b) of Azoles Used in Human Medicine and in Plant Protection Products (PPPs) with *S. cerevisiae* CYP51. **a** Chemical structures of selected clinical and agricultural azoles. **b** A – Voriconazole (PDB ID: 5HS1, pale blue carbon atoms), B – Difenoconazole (PDB ID: 5EAH, green), C – Itraconazole (PDB ID: 5EQB, forest green), D – Fluconazole (PDB ID: 4WMZ, yellow), E – ***S***-Tebuconazole (PDB ID: 5EAB, cyan)*, F – ***R***-Tebuconazole (PDB ID: 5EAC magenta). Protein is displayed as a white ribbon; heme cofactors are displayed in orange. Dark blue atoms are nitrogen, red – oxygen, light blue – fluorine, darker green – chlorine. Hydrogen bonds are shown as dashed yellow lines. *S-Tebuconazole is more active than ***R***-tebuconazole. Key water molecules and related hydrogen bonding networks were not observed in the tebuconazole structures due to their lower resolution compared to others. (Fig. 5b) shows binding modes of selected clinical azoles and PPPs in the co-crystal structures with *Saccharomyces cerevisiae* CYP51. All antifungal azoles display a common binding mode with the azole coordinating to the heme Fe ion. All compounds share a halogenated phenyl ring which binds in a hydrophobic pocket deep within the binding site. A water mediated hydrogen bonding network is seen between azoles containing a hydroxyl group and the protein (Y140 *S. cerevisiae* numbering, a key residue linked to resistance mutations) and the heme cofactor via water molecules. An additional hydrogen bonding network forms between the tail of the azoles and S382. Binding interactions between CYP51 and long tail azoles, such as itraconazole are predominantly hydrophobic but does include a similar water mediated hydrogen bond network to H381 and S382. Of note is the presence of either the hydroxyl group (e.g., voriconazole, fluconazole, tebuconazole, Fig. 5a) or a 5-member dioxolane ring (e.g., difenoconazole, itraconazole, propiconazole) where a hydrogen bonding network between the azole and Y140 is only seen with the hydroxyl group on the azole^171,172,276,277^. Created with ChemDraw®Professional 25.0, Revvity Signals Software, 2025.

Although the shared use of azoles across agriculture and medicine has been widely recognized, the emergence of cross-sectoral resistance is not merely a consequence of exposure frequency but reflects intrinsic structural constraints of azole–target interactions. Comparative analyzes of azole binding reveal that clinical triazoles and agricultural demethylation inhibitors engage highly conserved features of the CYP51 ligand-binding pocket, particularly for short-tailed compounds that rely on a limited set of water-mediated hydrogen bond interactions for stable inhibition. As a result, resistance-conferring substitutions that weaken these interactions can simultaneously reduce susceptibility to azoles deployed across sectors, even in the absence of direct drug exposure. This constrained molecular landscape explains the recurrent, independent selection of analogous resistance mechanisms across plant, environmental, and human pathogenic fungi. While the general mechanisms of CYP51 target modification and overexpression recur because all azoles share the same target enzyme, the specific resistance mutations themselves and their combinations are highly diverse across fungal taxa^14,157,166^. Figure 5 compares clinical azoles and PPPs, highlighting the structural similarities and differences for cross-resistance (Supplementary Table 4).

Azole responses in fungi can be described along two related but distinct dimensions: resistance versus tolerance, which describe the phenotypic response to antifungal exposure, and intrinsic versus acquired susceptibility, which refer to whether reduced susceptibility is a natural species trait or arises through genetic adaptation. Resistance typically reflects genetic alterations that increase MIC values, whereas tolerance represents a transient ability of fungal cells to survive azole exposure above the minimal inhibitory concentration (MIC) without genomic mutations. Tolerance is typically mediated by stress-response pathways, metabolic adjustments, or the formation of persister subpopulations, enabling temporary survival without fixed mutations. In contrast, resistance reflects permanent genetic alterations, such as point mutations or promoter changes in *CYP51A*, *ERG* genes, or *HMG1*, that result in persistently reduced drug susceptibility. Importantly, tolerant populations can act as precursors for the eventual emergence of resistant lineages under continuous azole pressure. The principal molecular mechanisms of resistance involve mutations or overexpression of *CYP51* genes encoding sterol 14α-demethylase, the primary azole target. Point mutations in CYP51 typically reduce azole binding to the enzyme active site, while promoter tandem repeats increase CYP51 expression and enzyme abundance. In parallel, ABC and MFS efflux transporters decrease intracellular azole concentrations and contribute to multidrug resistance phenotypes. In *A. fumigatus*, CYP51A mutations, such as TR_34_/L98H, TR_46_/Y121F/T289A, TR_53_ and others increase protein expression and reduce drug binding at the enzyme’s active site. In *Candida* and *Ca. auris*, CYP51 substitutions (encoded by *ERG11*) such as Y132F and K143R, coupled with ABC transporters (e.g., CDR1, CDR2) and MFS transporters (e.g., MDR1), drive multidrug resistance. Additional mutations in *ERG3*, *ERG6*, and *ERG11* alter ergosterol intermediates, while changes in *HMG1*, which regulates the mevalonate pathway, have similarly been associated with altered sterol intermediates and reduced azole susceptibility in both *Aspergillus* and *Candida*, linking upstream metabolic control to ergosterol biosynthesis balance. Dimorphic fungi show less-defined mechanisms, likely involving CYP51 sequence diversity, altered sterol composition, and azole transport regulation. Intrinsic resistance in *Fusarium* is linked to structural divergence in CYP51 paralogs (CYP51A, B, C), which collectively reduce azole binding. Similarly, *Scedosporium* species exhibit resistance through CYP51 mutations, upregulated efflux transporter activity, and biofilm formation, which further shields fungal cells from antifungal exposure. Beyond specific mutations, susceptibility and resistance are also shaped by the structural features of CYP51 itself: substitutions in regions, such as the BC-loop and I-helix influence how short-, medium-, and long-tailed azoles interact with the active site in plants, algae, and fungi. These same structural determinants underlie the selectivity of medical azoles against fungal versus human CYP51 and hepatic drug-metabolizing enzymes. Recent tetrazoles, such as oteseconazole, were designed to reduce heme iron interactions and improve fungal selectivity by exploiting the substrate entry channel. However, resistance has already emerged, illustrating that even these design advances cannot fully overcome the evolutionary plasticity of fungal CYP51^92,167–172^.

### Risk of dual use of compounds with the same MoA

**Fig. 6:**
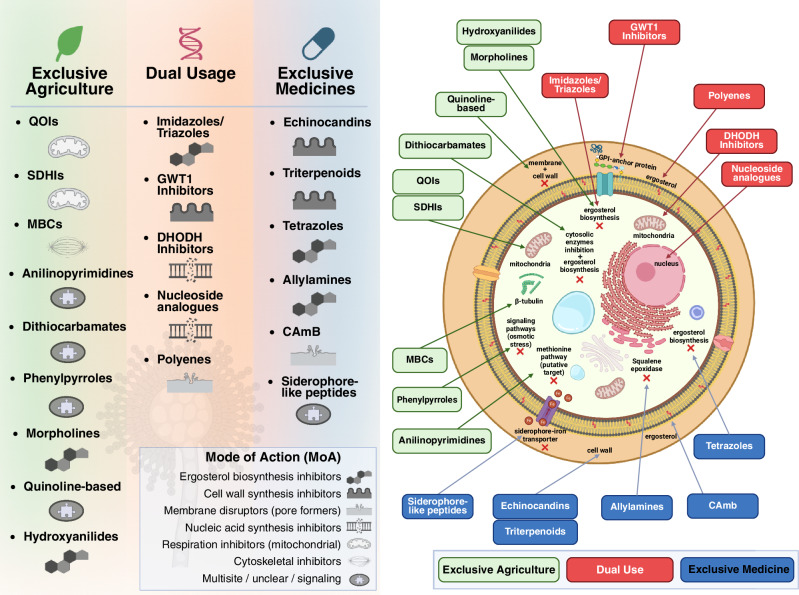
Antifungal drug classes by sector of use and mode of action. This figure categorizes antifungal compounds by their application: those used exclusively in agriculture (green), those shared between agricultural and medical settings (red), and those restricted to clinical use (blue). Each class is labeled with its primary molecular target and mode of action for clarity. Two leading antifungal candidates in clinical development, olorofim and fosmanogepix (APX001), exemplify growing concerns about dual-use applications. Olorofim, developed by F2G Ltd., inhibits dihydroorotate dehydrogenase (DHODH), a key enzyme in fungal pyrimidine biosynthesis. By disrupting fungal DNA and RNA synthesis, olorofim is particularly effective against molds like *Aspergillus*, thermally dimorphic fungi, and rare pathogens, such as *Scedosporium*, *Fusarium,* and *Neocosmospora*. Its agricultural counterpart, ipflufenoquin, developed by Nippon Soda Co., Ltd., targets the same enzymatic pathway for crop protection. However, it is not effective against *Candida* species^278–281^. Fosmanogepix, a prodrug of manogepix under development by Pfizer, inhibits Gwt1, an enzyme essential for glycosylphosphatidylinositol (GPI) anchor biosynthesis. By disrupting fungal cell wall structure, fosmanogepix is effective against pathogens like *Candida*, *Aspergillus*, *Fusarium* and *Neocosmospora*. In agriculture, aminopyrifen, developed by Agro-Kanesho Co., Ltd., shares a similar target profile and is used for fungal disease control in plants. These overlaps in antifungal mechanisms highlight the potential for resistance evolution across sectors^281–284^. Additional context is provided in refs. ^10,92,217,275,285–288^. Created in BioRender. ROOHI, B. (2026) https://BioRender.com/4yxfg4i.

Recent antifungal developments, such as olorofim and fosmanogepix, currently in clinical trials, have inspired structurally and mechanistically similar compounds now used in agriculture (see Fig. 6 legend for details on companies and targets). This overlap stresses growing concerns about antifungal dual-use and its implications for cross-resistance.

While the dual use of triazoles has already demonstrated global consequences for resistance in human pathogenic molds and yeasts, similar risks are emerging with novel modes of action. In 2023, the Australian Pesticides and Veterinary Medicines Authority (APVMA) approved Ipflufenoquin (DHODH inhibitors [FRAC 52]) for controlling *Botrytis cinerea*, the causal agent of gray mold, in strawberries. The US Environmental Protection Agency (EPA) has also approved ipflufenoquin for use in fruit crops. Due to its structural and mechanistic similarity to the clinical antifungal olorofim, this decision raised alarm within the medical mycology community. Infectious disease specialists and mycology experts in Australia and New Zealand issued a joint statement urging its suspension, citing the risk of cross-resistance and the potential threat to the long-term efficacy of critical antifungal agents in human medicine^8,173,174^. The agricultural use of Ipflufenoquin could therefore select for DHODH mutations that undermine the future clinical utility of olorofim. Despite these concerns, ipflufenoquin remains approved and in use in Australia.

Current fungicide authorization processes do not account for activity against human pathogenic fungi, such as *A. fumigatus*. The emergence of azole-resistant *A. fumigatus* has prompted agencies like the Environmental Protection Agency (EPA) and the European Food Safety Agency (EFSA) to develop conceptual frameworks for assessing the risks of fungicides with activity against human pathogens^4,175^.

### Solutions: current challenges and management strategies

While the solutions outlined in Table 2 provide targeted measures across governance, innovation, surveillance, agriculture, regulation, technology, and sustainability, their effectiveness will depend on coordinated implementation, equitable access, and sustained political will. Disparities in diagnostic capacity, antifungal availability, and agricultural alternatives risk widening the resistance gap between high- and low-resource settings, which is likely to accelerate the global spread of resistant pathogens. In addition, current surveillance is largely reactive, identifying outbreaks only after cross-sectoral transmission has occurred, rather than predictive, which would require large-scale environmental and genomic monitoring. Integrating antifungal resistance into National Action Plans (NAPs) for antimicrobial resistance (AMR) and leveraging the Quadripartite Alliance on One Health, comprising the World Health Organization (WHO), the Food and Agriculture Organization of the United Nations (FAO), the World Organization for Animal Health (WOAH), and the United Nations Environment Program (UNEP), can provide a structured framework for global action.

**Table 2:** Integrated One Health Strategies to Combat Antifungal Resistance: Challenges, Solutions, and Expected Impact

Category	Key Challenges	Proposed Solutions & Rationale	References
**Global governance**	No unified international framework for antifungal stewardship; weak enforcement in LMICs	Develop and enforce global antifungal use standards via WHO, FAO, WOAH, UNEP. Integrate antifungal resistance into AMR National Action Plans. Coordinate cross-border regulations to control fungicide trade and misuse. Impact: Ensures consistent global policies, reduces loopholes in fungicide supply chains.	^92,214–216^
**Antifungal R&D**	Limited drug classes; slow innovation; high costs; unequal access	Launch public–private partnerships to share R&D risks and costs. Adapt models like CARB-X for antifungal innovation. Incentivize development of drugs with novel mechanisms and agriculture–medicine separation. Impact: Expands treatment options and reduces cross-resistance risks.	^2,92,217,218^
**Surveillance & monitoring**	Patchy resistance data; lack of environmental surveillance	Expand WHO GLASS to include fungal pathogens. Standardize lab methods and resistance reporting. Implement routine testing of soils, water, and crops for resistant fungi. Impact: Identifies hotspots early, informs targeted interventions.	^168,219^
**Agricultural practices**	Overreliance on azoles; limited availability and adoption of effective alternatives	Promote IPM combining biological, mechanical, and minimal chemical use. Rotate fungicide subclasses annually to delay resistance. Provide financial incentives for sustainable farming. Impact:Reduces environmental azole load while maintaining crop yields.	^220–224^
**Regulatory measures**	Unrestricted OTC fungicide sales; weak residue control	Mandate prescriptions for high-risk fungicides. Enforce residue limits on food. Adapt proven antibiotic policy models, e.g., EU ban on growth promoters. Impact: Cuts unnecessary exposure, lowers selection pressure.	^92,225–227^
**Technological advancements**	Limited diagnostics in low-resource areas; lack of precision tools	Deploy portable diagnostics for rapid detection. Use bioinformatics to track resistance genes and guide drug use. Implement precision agriculture to target fungicide application. Impact: Enables smarter, data-driven stewardship.	^228,229^
**Sustainable practices**	Persistent azole residues in environment; biodiversity loss	Apply bioremediation to degrade fungicides in soils/water. Restore habitats to rebuild ecological resilience. Support crop diversification to reduce fungicide dependency. Impact: Protects ecosystems and long-term agricultural viability.	^92,230^
**One Health approach**	Lack of cross-sector collaboration and public awareness	Integrate human, animal, and plant health policies under One Health. Run global awareness campaigns targeting farmers, clinicians, policymakers. Include antifungal stewardship in professional training curricula. Impact: Aligns health and agriculture goals, fosters sustainable practices.	^92,228,230^

* *WHO* World Health Organization, *FAO* Food and Agriculture Organization, *WOAH* World Organization for Animal Health, *UNEP* United Nations Environment Program, *LMIC* Low- and Middle- Income Countries, *CARB-X* Combating Antibiotic-Resistant Bacteria Biopharmaceutical Accelerator, *WHO GLASS* WHO Global Antimicrobial Resistance and Use Surveillance System, *IPM* Integrated Pest Management, *OTC* Over-the-counter.

The rise of antifungal resistance emphasizes a fundamental truth: biological systems do not operate in isolation. The forces driving resistance in hospitals are also shaped by environmental and agricultural practices, and our ability to address this challenge will depend on science and our capacity to recognize and mitigate the interconnected pressures we impose on microbial evolution. Confronting this reality through knowledge-based azole design and stewardship within a One Health framework is essential. Ignoring this opportunity will negatively shape the trajectory of global health for decades to come.

### Literature search strategy

Relevant publications were identified through systematic searches of PubMed and Web of Science using search terms, such as *azole resistance*, *One Health*, *antifungal and fungicide use in agriculture, fungicide resistance, azole contamination*, *cross-sectoral resistance*, *fungal pathogens in crops and animals*, and *human fungal infections*. Priority was given to peer-reviewed, English-language studies published in the past decade, while seminal earlier works were included to provide historical context. All authors reviewed and revised all outputs as needed and take full responsibility for the accuracy and integrity of the content.

## Supplementary information

Supplementary Information
