# Supplementary material for: The dual role of azoles: lifesaving antifungals and drivers of resistance – a One Health perspective
Source: Nat Commun. 2026 Apr 19;17:5407. doi: 10.1038/s41467-026-71762-9 (PMC13280150; doi:10.1038/s41467-026-71762-9)
Supplement: Supplementary file 1 — Supplementary Information [file 41467_2026_71762_MOESM1_ESM.pdf]

Supplementary Table 1. FRAC classification and stewardship notes for fungicide classes and representative actives.
Fungicide classes are grouped according to FRAC mode-of-action codes, with representative active ingredients, primary molecular targets, resistance risk, and stewardship recommendations. Classification and stewardship guidance are based on FRAC Code List 2024/2025 and FRAC resistance management guidelines.

Fungicide class	REPRESENTATIVE ACTIVES	FRAC CODE	Primary molecular target	Resistance risk	STEWARDSHIP NOTES
**Azoles (DMIs)**	Tebuconazole; Prothioconazole; Difenoconazole; Propiconazole; Imazalil	3	CYP51 (sterol 14α-demethylase)	Medium	Rotate FRAC codes; use mixtures; limit applications per season; avoid repeated solo curative sprays
**QoIs (strobilurins)**	Azoxystrobin; Pyraclostrobin	11	Qo site of cytochrome bc_1 complex (complex III)	High	Apply only in mixtures; rotate with non-QoI MoAs; limit sequential applications
**SDHIs**	Fluxapyroxad; Boscalid	7	Succinate dehydrogenase (complex II)	High	Use in mixtures; rotate MoAs; monitor sensitivity shifts
**QiIs**	Cyazofamid; Amisulbrom	21	Qi site of cytochrome bc_1 complex (complex III)	High (primarily oomycetes)	Use only in mixtures with different MoAs; adhere strictly to label limits
**MBCs (benzimidazoles)**	Carbendazim; Thiophanate-methyl	1	β-tubulin	High	Restrict use to populations with documented sensitivity; avoid repeated applications
**Multi-site protectants**	Copper compounds (M1); Sulfur (M2); Mancozeb (M3); Chlorothalonil (M5)	M1–M5	Multiple cellular targets	Low	Use as mixture backbone; consider environmental and regulatory constraints (e.g., copper accumulation)
**DHODH inhibitors (quinazolinones)**	Ipflufenoquin	U17	Dihydroorotate dehydrogenase	Emerging / unknown	Treat as new single-site MoA; use mixtures; restrict total applications; actively monitor sensitivity

Supplementary Table 2. Discovery timelines, domains of use, and references for major antifungal agents. The table summarizes key antifungal compounds, their chemical class, first reported year of development or discovery, and whether their primary domain of use is clinical or agricultural.

ANTIFUNGAL AGENT	DRUG CLASS/TYPE	DOMAIN OF USE	APPROXIMATE YEAR OF DISCOVERY/DEVELOPMENT	KEY REFERENCE
**NYSTATIN**	Polyene	Clinical	1950	[3] Sousa et al., 2023
**AMPHOTERICIN B**	Polyene	Clinical	1953	[4] Dutcher et al., 1968
**KETOCONAZOLE**	Imidazole	Clinical	1977	[5] Fischr , Gangellin, 2006
**MICONAZOLE**	Imidazole	Clinical	1969	[6] Fothergill, 2014
**ITRACONAZOLE**	Triazole	Clinical	1978	[5] Fischr, Gangellin, 2006
**FLUCONAZOLE**	Triazole	Clinical	1981	[5] Fischr, Gangellin, 2006
**TERCONAZOLE**	Triazole	Clinical	1983	[7] Heeres et al., 1983
**POSACONAZOLE**	Triazole	Clinical	2004	[8] Drugs.com, 2004
**VORICONAZOLE**	Triazole	Clinical	1990	[5] Fischr, Gangellin, 2006
**ISAVUCONAZOLE**	Triazole	Clinical	2010	[23] Thompson, 2010
**IBREXAFUNGERP**	Triterpenoid / Glucan synthase inhibitor	Clinical	2013	[9] Ghannoum et al., 2020
**OPELCONAZOLE**	Triazole	Clinical	2013	[19] Murray et al., 2020
**OTESECONAZOLE**	Tetrazole	Clinical	2013	[20] Vanreppelen et al,. 2023
**REZAFUNGIN**	Echinocandin	Clinical	2016	[10] Thompson et al., 2022
**FOSMANOGEPIX**	Gwt1 Inhibitor	Clinical	2015	[11] Almajid et al., 2023
**OLOROFIM**	DHODH Inhibitor	Clinical	2015	[12] Wiederhold, 2020
**VL-2397**	Siderophore-like peptide	Clinical	2015	[18] Shaw, 2022
**CAMB**	Polyene (Cochleated)	Clinical	2015	[21] Aigner, 2020
**ATI-2307**	Arylamidine / Mitochondrial inhibitor	Clinical	2019	[22] Cui et al., 2022
**MANCOZEB**	Dithiocarbamate	Agricultural	1961	[24] Gullino et al., 2010
**BENOMYL**	Benzimidazole	Agricultural	1968	[25] Pearson & Miller, 2014
**PROCHLORAZ**	Imidazole	Agricultural	1974	[13] Bayer CropScience AG. 2009
**CARBENDAZIM**	Benzimidazole	Agricultural	1973	[14] University of Hertfordshire
**PROPICONAZOLE**	Triazole	Agricultural	1979	[26] PubChem
**TEBUCONAZOLE**	Triazole	Agricultural	1986	[27] Dong et al., 2024
**DIFENOCONAZOLE**	Triazole	Agricultural	1989	[28] HB Plant Protection
**EPOXICONAZOLE**	Triazole	Agricultural	1990	[29] Wendeborn et al., 2012
**BOSCALID**	SDHI	Agricultural	2003	[15] EPA Registration
**PROTHIOCONAZOLE**	Triazole	Agricultural	2003	[30] EPA Registration
**MEFENTRIFLUCONAZOLE**	Triazole	Agricultural	2019	[16] Umetsu, Shirai, 2020
**AMINOPYRIFEN**	Unknown / Novel mode of action	Agricultural	2017	[17] Hatamoto et al., 2019
**IPFLUFENOQUIN**	Quinoline	Agricultural	2017	[31] ChemicalBook, 2024

Supplementary Table 3. Environmental concentrations of azole compounds in relation to toxicological and resistance-selection thresholds.

Notes on quantitative interpretation

- Resistance selection can occur at concentrations below MIC, often described by the minimal selective concentration, with $0 < MSC < MIC$; environmental $ng \cdot L^{-1}$–$\mu g \cdot L^{-1}$ (waters) and $\mu g \cdot kg^{-1}$–$mg \cdot kg^{-1}$ (soils/compost) bands can be selection-relevant even when far below mammalian toxicology thresholds [7,4].
- EFSA/ECHA toxicology thresholds (NOAEL, RfD, DNEL) for azole DMIs are typically orders of magnitude above environmental residues, underscoring that resistance risk management requires selection-based metrics rather than reliance on human toxicology lines [34,35,38].

ENVIRONMENTAL SETTING	AZOLE COMPOUND(S)	USE SECTOR	MEASURED CONCENTRATION RANGE	EFFECT ENDPOINT	EFFECT THRESHOLD (CONTEXT)	APPROXIMATE C / THRESHOLD RATIO	INTERPRETATION	KEY REFERENCES
**FLOWER-BULB WASTE AND COMPOST (NETHERLANDS)**	Tebuconazole; Propiconazole; Epoxiconazole	Agricultural (DMI fungicides)	$\mu g \cdot kg^{-1}$–$mg \cdot kg^{-1}$ in waste streams and compost piles	Selection of azole-resistant *Aspergillus fumigatus* (e.g. TR34/L98H) during thermophilic composting	Resistance selection observed at $mg \cdot kg^{-1}$ order under composting conditions	≈ 0.3 to >1 (site-dependent)	Environmental residues overlap or exceed resistance-selection–relevant concentrations despite being far below mammalian toxicity thresholds	[32]Schoustra et al., 2019; [33]Verweij et al., 2016
**AGRICULTURAL SOILS AFTER LABELLED USE (EU MONITORING)**	Tebuconazole; Propiconazole; Difenoconazole	Agricultural (DMI fungicides)	Typically 10–500 $\mu g \cdot kg^{-1}$ after application (substance- and soil-dependent)	Enrichment of resistant environmental *A. fumigatus* under repeated sub-MIC exposure	Selection plausible within MSC band below MIC; below clinical MICs but resistance-relevant	≈ 0.2–1 (context-dependent)	Residues are below NOAELs and clinical MICs but fall within concentrations compatible with resistance selection	[34]EFSA tebuconazole 2014; [35]EFSA propiconazole 2011; [33]Verweij et al., 2016; [38]Andersson & Hughes, 2014
**WASTEWATER EFFLUENTS AND RECEIVING SURFACE WATERS**	Fluconazole; Itraconazole; Voriconazole	Medical	$ng \cdot L^{-1}$–$\mu g \cdot L^{-1}$ (influent and effluent; European surveys)	Resistance selection in aquatic biofilms and sediments under prolonged exposure	Minimal selective concentration (MSC) below MIC	Non-zero fraction of MSC (qualitative)	Concentrations are far below therapeutic or toxic doses yet compatible with resistance-selection frameworks	[36]Aus der Beek et al., 2016; [37]Santos et al., 2013; [38]Andersson & Hughes, 2014
**GREEN WASTE AND MUNICIPAL ORGANIC RECYCLING STREAMS**	Tebuconazole; Epoxiconazole; Propiconazole	Agricultural (mixed triazole DMIs)	$\mu g \cdot kg^{-1}$–$mg \cdot kg^{-1}$ in feedstock and compost	TR-mediated *A. fumigatus* resistance selection during waste handling	$mg \cdot kg^{-1}$ order supports selection under thermophilic composting	≈ 0.5 to >1	Recurrent overlap between contamination levels and resistance-selection thresholds in waste-processing environments	[32]Schoustra et al., 2019; [33]Verweij et al., 2016

Supplementary Table 4. Comparative Analysis of Azoles Used in Human Medicine and in Plant Protection Products (PPPs)
Comparative analysis of resistance-associated mutations in *CYP51* across azoles used in human medicine and agriculture. Representative examples are shown for clinical azoles (e.g., voriconazole, itraconazole, fluconazole, isavuconazole) and plant protection products (e.g., difenoconazole, propiconazole, tebuconazole, mefentrifluconazole).

ANTIFUNGAL	CYP51-LINKED RESISTANCE MUTATIONS (REPRESENTATIVE EXAMPLES)
**VORICONAZOLE (CLINICAL)**	TR34/L98H in *Aspergillus fumigatus* [39]
**DIFENOCONAZOLE (AGRICULTURAL)**	CYP51 mutations in *Colletotrichum gloeosporioides* [40]
**ITRACONAZOLE (CLINICAL)**	G448S in *Aspergillus fumigatus* [41]
**PROPICONAZOLE (AGRICULTURAL)**	Y464S in *Cercospora beticola* [42]
**FLUCONAZOLE (CLINICAL)**	G484S in *Cryptococcus neoformans* [43]
**TEBUCONAZOLE (AGRICULTURAL)**	G461S in *Monilinia fructicola* [44]
**ISAVUCONAZOLE (CLINICAL)**	G448S in *Aspergillus fumigatus* [41]
**MEFENTRIFLUCONAZOLE (AGRICULTURAL)**	L144F in *Fusarium pseudograminearum* [45]

Supplementary references

1 Fungicide Resistance Action Committee (FRAC). *FRAC Code List 2024/2025: Fungicides sorted by mode of action.* FRAC (2024).

2 Fungicide Resistance Action Committee (FRAC). *Fungicide resistance management guidelines.* FRAC (2024).

3 Sousa, F, Nascimento, C., Ferreira, D., Reis, S., & Costa, P. Reviving the interest in the versatile drug nystatin: a multitude of strategies to increase its potential as an effective and safe antifungal agent. *Adv. Drug Deliv. Rev.* **200**, 114969 (2023).

4 Dutcher, J. D. The discovery and development of amphotericin B. *J. Antibiot.* **54**(Suppl.), 296–298 (1968).

5 Fischer, J. & Ganellin, C. R. *Analogue-based drug discovery*. IUPAC, 503 (2006).

6 Fothergill, A. W. Miconazole: a historical perspective. *Expert Rev. Anti Infect. Ther.* **4**(2), 171–175 (2014).

7 Heeres, J., Hendrickx, R. & Van Cutsem, J. Antimycotic azoles. 6. Synthesis and antifungal properties of terconazole, a novel triazole ketal. *J. Med. Chem.* **26**(4), 611–613 (1983).

8 Drugs.com. *Noxafil (posaconazole) FDA approval history*. Available at: https://www.drugs.com/history/noxafil.html(Accessed: 20 March 2025).

9 Ghannoum, M. *et al.* Ibrexafungerp: a novel oral triterpenoid antifungal in development for the treatment of *Candida auris* infections. *Antibiotics* **9**(9), 539 (2020).

10 Thompson, G. R. 3rd *et al.* Rezafungin versus caspofungin for treatment of candidaemia and invasive candidiasis (ReSTORE): a multicentre, double-blind, double-dummy, randomised phase 3 trial. *Lancet* **400**(10367), 2073–2084 (2022).

11 Almajid, A. *et al.* Fosmanogepix: the novel antifungal agent's comprehensive review of in vitro, in vivo, and current insights from advancing clinical trials. *Cureus* **15**(8), e43866 (2023).

12 Wiederhold, N. Review of the novel investigational antifungal olorofim. *J. Fungi* **6**(3), 122 (2020).

13 Bayer CropScience AG. *The Pesticide Manual*. 16th edn. BCPC (2009).

14 University of Hertfordshire. *Carbendazim (Ref: BAS 346F). Pesticide Properties DataBase*. Available at: https://sitem.herts.ac.uk/aeru/iupac/Reports/116.htm (Accessed: 17 March 2025).

15 U.S. Environmental Protection Agency. *Pesticide fact sheet: Boscalid (PC Code: 128008)*. Available at: https://www3.epa.gov/pesticides/chem_search/reg_actions/registration/fs_PC-128008_01-Jul-03.pdf (Accessed: 17 March 2025).

16 Umetsu, N. & Shirai, Y. Development of novel pesticides in the 21st century. *J. Pestic. Sci.* **45**(2), 54–74 (2020).

17 Hatamoto, M., Aizawa, R., Kobayashi, Y., & Fujimura, M. A novel fungicide aminopyrifen inhibits GWT-1 protein in glycosylphosphatidylinositol-anchor biosynthesis in *Neurospora crassa*. *Pestic. Biochem. Physiol.* **157**, 140–146 (2019).

18 Shaw, K. J. Review of the novel siderophore-like antifungal agent for the treatment of invasive aspergillosis. *J. Fungi***8**(9), 922 (2022).

19 Murray, A. *et al.* A novel inhaled antifungal agent for the treatment of respiratory fungal infections. *J. Fungi* **6**(4), 373 (2020).

20 Vanreppelen, G. *et al.* Oteseconazole (VIVJOA) for prevention of recurrent vulvovaginal candidiasis. *Trends Pharmacol. Sci.***44**(1), 64–65 (2023).

21 Aigner, M. & Lass-Flörl, C. Encochleated amphotericin B: is the oral availability of amphotericin B finally reached? *J. Fungi* **6**(2), 66 (2020).

22 Cui, X., Wang, L., Lü, Y. & Yue, C. Development and research progress of anti-drug resistant fungal drugs. *J. Infect. Public Health* **15**(9), 986–1000 (2022).

23 Thompson, G. R. III & Wiederhold, N. P. Isavuconazole: a comprehensive review of spectrum of activity of a new triazole. *Mycopathologia* **170**, 291–313 (2010). https://doi.org/10.1007/s11046-010-9324-3

24 Gullino, M. L. *et al*. Mancozeb: past, present, and future. *Plant Dis.* **94**, 1076–1087 (2010).

25 Pearson, M. A. & Miller, G. W. Benomyl. In *Encyclopedia of Toxicology* (ed. Wexler, P.) 411–412 (Elsevier, 2014).

26 National Center for Biotechnology Information. PubChem Compound Summary for CID 51086, Propiconazole.
Available at: https://pubchem.ncbi.nlm.nih.gov/compound/Propiconazole (Accessed: 19 February 2025).

27 Dong, B. A comprehensive review on toxicological mechanisms and transformation products of tebuconazole:

insights on pesticide management. *Sci. Total Environ.* **908**, 168264 (2024).

28 HB Plant Protection. *Difenoconazole: an effective treatment for fungal infections*. Available at: https://www.hb-p.com/article/difenoconazole-an-effective-treatment-for-fungal-infections/ (Accessed: 19 February 2025).

29 Wendeborn, S., Godineau, E., Mondière, R., Smejkal, T. & Smits, H. Chirality in agrochemicals. In *Comprehensive Chirality* (eds Carreira, E. M. & Yamamoto, H.) **1**, 120–166 (Elsevier, 2012). https://doi.org/10.1016/B978-0-08-095167-6.00102-6

30 U.S. Environmental Protection Agency. *Registration of Prothioconazole*. Available at: https://www3.epa.gov/pesticides/chem_search/reg_actions/registration/fs_PC-113961_14-Mar-07.pdf (Accessed: 19 February 2025).

31 ChemicalBook. What kind of fungicide is ipflufenoquin? *ChemicalBook*. https://www.chemicalbook.com/article/what-kind-of-fungicide-is-ipflufenoquin.htm (accessed 25 Mar 2025).

32 Schoustra, S. E. *et al*. Environmental hotspots for azole resistance selection of *Aspergillus fumigatus* in the Netherlands. *Emerging Infectious Diseases,* **25**(7):1347-1353 (2019). https://doi.org/10.3201/eid2507.181625

33 Verweij, P. E., Snelders, E., Kema, G. H. J., Mellado, E. & Melchers, W. J. G**.** Azole resistance in *Aspergillus fumigatus*: a side-effect of environmental fungicide use? *The Lancet. Infectious diseases*, **9**(12), 789–795 (2009). https://doi.org/10.1016/s1473-3099(09)70265-8

34 European Food Safety Authority (EFSA). Conclusion on the peer review of the pesticide risk assessment of the active substance tebuconazole. *EFSA J.* **12**, 3480 (2014). https://doi.org/10.2903/j.efsa.2014.3485

35 European Food Safety Authority (EFSA). Peer review of the pesticide risk assessment of the active substance propiconazole. *EFSA J.* **15**, 4887 (2017). https://doi.org/10.2903/j.efsa.2017.4887

36 Aus der Beek, T. *et al.* Pharmaceuticals in the environment—global occurrences and perspectives. *Environ. Toxicol. Chem.* **35**, 823–835 (2016). https://doi.org/10.1002/etc.3339

37 Santos, L. H. M. L. M. *et al*. Ecotoxicological aspects related to the presence of pharmaceuticals in the aquatic environment. *J. Hazard. Mater.* **175**, 45–95 (2010). https://doi.org/10.1016/j.jhazmat.2009.10.100

38 Andersson, D. I. & Hughes, D. Microbiological effects of sublethal levels of antibiotics. *Nat. Rev. Microbiol.* **12**, 465–478 (2014). https://doi.org/10.1038/nrmicro3270

39 Hosseini, S. *et al.* The Molecular Basis of the Intrinsic and Acquired Resistance to Azole Antifungals in *Aspergillus fumigatus*. *J. Fungi* **10**(12), 820 (2024). https://doi.org/10.3390/jof10120820

40 Wei, L.-L. *et al*. Mutations and Overexpression of CYP51 Associated with DMI-Resistance in *Colletotrichum gloeosporioides* from Chili. *Plant Dis.* **104**, 1628–1636 (2020). https://doi.org/10.1094/PDIS-08-19-1628-RE

41 Gonzalez-Jimenez, I. *et al.* A Cyp51B mutation contributes to azole resistance in *Aspergillus fumigatus*. *J. Fungi* **6**, 315 (2020). https://doi.org/10.3390/jof6040315

42 Kumar, R. *et al.* Characterization of the molecular mechanisms of resistance against DMI fungicides in *Cercospora beticola* populations from the Czech Republic. *J. Fungi* **7**, 1062 (2021). https://doi.org/10.3390/jof7121062

43 Zhang, J. *et al*. The fungal CYP51s: their functions, structures, related drug resistance, and inhibitors. *Front. Microbiol.* **10**, 691 (2019).https://doi.org/10.3389/fmicb.2019.00691

44 Lichtemberg, P. S. *et al.* The point mutation G461S in the *Mf*CYP51 gene is associated with tebuconazole resistance in *Monilinia fructicola* populations in Brazil. *Phytopathology* **107**, 1507–1514 (2017). https://doi.org/10.1094/PHYTO-02-17-0050-R

45 Li, H. *et al.* Mefentrifluconazole resistance risk and resistance-related point mutation in FpCYP51B of *Fusarium pseudograminearum*. *J. Agric. Food Chem.* **71**, 18014–18023 (2023). https://doi.org/10.1021/acs.jafc.3c08014
